# On the origin of the genetic variation in infectious disease prevalence: Genetic analysis of disease status versus infections for Digital Dermatitis in Dutch dairy cattle

**DOI:** 10.1111/jbg.12635

**Published:** 2021-06-09

**Authors:** Pranav Shrikant Kulkarni, Floor Biemans, Mart C.M. de Jong, Piter Bijma

**Affiliations:** ^1^ Animal Breeding and Genomics Wageningen University & Research Wageningen The Netherlands; ^2^ Wageningen Business Economics Group Wageningen University & Research Wageningen The Netherlands; ^3^ Farm Animal Health Faculty of Vet. Med Department of Population Health Sciences Utrecht University Utrecht The Netherlands; ^4^ Quantitative Veterinary Epidemiology Wageningen University & Research Wageningen The Netherlands; ^5^ Centre for Veterinary Epidemiology and Risk Analysis UCD School of Veterinary Medicine University College Dublin Belfield, Dublin Ireland; ^6^ INRAE BIOEPAR Nantes France

**Keywords:** Mortellaro, heritability, susceptibility, recovery, disease transmission

## Abstract

The purpose of this study was to investigate the origin of the genetic variation in the prevalence of bovine digital dermatitis (DD) by comparing a genetic analysis of infection events to a genetic analysis of disease status. DD is an important endemic infectious disease affecting the claws of cattle. For disease status, we analysed binary data on individual disease status (0,1; indicating being free versus infected), whereas for infections, we analysed binary data on disease transmission events (1,0; indicating becoming infected or not). The analyses of the two traits were compared using cross‐validation. The analysis of disease status captures a combination of genetic variation in disease susceptibility and the ability of individuals to recover, whereas the analysis of infections captures genetic variation in susceptibility only. Estimated genetic variances for both traits indicated substantial genetic variation. The GEBV for disease status and infections correlated with only 0.60, indicating that both models indeed capture distinct information. Together, these results suggest the presence of genetic variation not only in disease susceptibility, but also in the ability of individuals to recover from DD. We argue that the presence of genetic variation in recovery implies that breeders should distinguish between infected individuals versus infectious individuals. This is because epidemiological theory shows that selection for recovery is effective only when it targets recovery from being infectious.

## 1 INTRODUCTION

Infectious diseases not only reduce the productivity of farm animals, but also cause considerable losses related to disease control and cure measures (Ifende et al., 2014). Next to disease control measures such as vaccination and treatment, genetic selection of the host population can be used to combat infectious diseases in livestock (Davies et al., 2009; Deb et al., 2012; Jovanović et al., 2009). Many studies on host resistance or susceptibility to particular diseases have shown that host genetic variance can be successfully manipulated by use of selection and breeding techniques (Bishop & MacKenzie, 2003; Bishop & Stear, 2003; Guy et al., 2009; Neibergs et al., 2014; Råberg et al., 2007). Anacleto et al. (2015) argue that selection and breeding are a proactive preventive measure, which may be longer in duration of effect and in the long term less economically eventful than other interventions. Thus, in addition to measures such as vaccination and treatment, genetic selection is an important tool to combat infectious diseases in livestock (and plants).

Disease traits in livestock are typically recorded as a binary disease status of an individual, indicating absence (0, non‐infected) or presence (1, infected) of the disease in the individual (Gianola, 1982). Because the population average value of individual disease status is equal to the prevalence of the disease in the population (*i.e*. the fraction of individuals having the disease), models of binary disease status implicitly also address the prevalence of the disease. In applied animal breeding, disease status data are often analysed using simple linear mixed models, where the binary record of the individual is linearly related to its breeding value. Generalized linear mixed models, such as threshold models, are also used and are statistically much more appropriate (Gianola, 1982), but benefits over the simpler linear models are often found to be small in practice especially without adequate Bayesian priors (Hadfield & Nakagawa, 2010; Sorensen & Gianola, 2007).

However, neither linear models nor the commonly used generalized linear models consider the underlying dynamics of the transmission of infectious diseases in the population (Diekmann et al., 2012). Infectious diseases are fundamentally different from non‐communicable diseases, because the probability that an individual becomes infected depends on the other individuals in the herd. As a consequence, the prevalence of an infectious disease in a group of individuals is a property of the entire group. For this reason, epidemiologists believe that it is essential to consider the transmission dynamics when the aim is to predict the consequences of interventions, such as vaccination (De Jong & Kimman, 1994) or genetic selection (Anche et al., 2015; Bishop & Woolliams, 2014; Diekmann & Heesterbeek, 2000; Nieuwhof et al., 2009).

### 1.1 A sketch of the objective

The fact that infectious disease prevalence depends on the transmission dynamics in the population raises the question of how to interpret the breeding value of a traditional linear model applied to individual disease status (0/1; disease status model, DSM) which ignores these transmission dynamics. For example, whether such a breeding value would be different from a breeding value based on epidemiological models, and whether or not it is predictive of response to selection (Hulst et al., 2021). This study is a step towards answering that question. Throughout, we will ignore variation among individuals in “infectivity”, *that is* the propensity of individuals to infect others, which is beyond the scope of this manuscript.

In the Appendix S1, we summarize some basic epidemiological theory. This appendix shows that the prevalence of an endemic infectious disease is determined by the basic reproduction number (*R*_0_). The *R*_0_ depends on the susceptibility of individuals to become infected, and on their ability to recover from being *infectious*. Note, we allow for a distinction between being *infected* (*i.e*. having disease status 1) versus. being *infectious* (being able to infect others). Because a disease status of 1 usually means that an individual is infected, the breeding value of a DSM is a function of both the susceptibility of the individual, and of its ability to recover from being *infected* (See also Bijma, 2020; Eqn 4.4). Hence, disease prevalence depends on the rate at which individuals recover from being infectious, whereas the usual breeding value from the DSM depends on the rate at which individuals recover from being infected. Hence, there is a discrepancy between common breeding values and the epidemiological factors that determine prevalence. (In the Appendix S2, we provide a numerical example showing that the prevalence is unaffected when individuals recover sooner from an infected, but non‐infectious, disease status, as is well‐known in epidemiology especially in case of seasonality (Aron & Schwartz, 1984; Dietz, 1976) and vaccination (Anderson & May, 1982)).

The distinction between infected versus infectious disease statuses, as discussed in the previous paragraph, would be irrelevant for livestock genetic improvement when recovery would show no genetic variation. In other words, if the full heritable variation in binary disease status would originate solely from genetic variation in susceptibility, then there would be no need to distinguish between infected versus infectious individuals, and an analysis of disease status may suffice (apart from other issues such as infectivity, and indirect genetic effects due to susceptibility; Anche et al., 2015; Bijma, 2020). Hence, to optimize genetic selection against infectious diseases, we need to know whether genetic variation in binary disease status originates solely from genetic differences in disease susceptibility, or whether other factors such as recovery also play a role.

To address the question of whether or not the full heritable variation in binary diseases status originates solely from genetic variation in susceptibility, we compare results of a conventional analysis of binary disease status to those of an analysis of infection events. For the analysis of binary disease status, we will use a linear mixed model, hereafter referred to as a disease status model (DSM). For the analysis of infection events, we will use a generalized linear mixed model, hereafter referred to as infection model (IM; following Biemans et al., 2019). Hence, in the IM, the dependent variable indicates whether (y = 1) or not (y = 0) a previously non‐infected individual has become infected in a time interval. The DSM captures genetic variation in both susceptibility and recovery (Bijma, 2020), while the IM captures genetic variation in susceptibility only. Thus, a difference between results of both models suggests the presence of genetic variation in recovery. To address this issue, we will use longitudinal data on digital dermatitis (DD), an endemic infectious claw disease in cattle, collected in Dutch herds of dairy cattle. Our focus is on the differences in estimates of genetic parameters and breeding values for both traits, and on the interpretation of those differences. Hence, our aim is not to find a superior statistical modelling strategy, but to understand the biological meaning of the differences between an analysis of disease status versus an analysis of infection events (i.e. disease transmission) which are two distinct traits.

### 1.2 Digital dermatitis

Biemans et al. (2018) developed a generalized linear mixed model for bovine digital dermatitis (DD). Bovine DD is an endemic infectious claw disease, found predominantly in the hind feet of dairy cattle and was first documented by Cheli and Mortellaro (1974). It can cause lameness and considerable losses in terms of milk production (Frankena et al., 1991; de Jesús Argáez‐Rodríguez et al., 1997; Rodríguez‐Lainz et al., 1996; Somers et al., 2005). Van der Linde et al. (2010) estimated the cost per case for DD to be € 68. Animals that recover from DD are immediately susceptible again, and the disease can recur numerous times. The main transmission route is via pathogens shed into the environment by infected animals. This transmission often goes undetected (Biemans et al., 2018).

Since DD status is heritable, the prevalence of DD can be reduced by selective breeding (Van der Linde et al., 2010; Van der Waaij et al., 2005). Heritability estimates for DD status (0/1) from linear‐threshold models and logistic models (liability heritability) range from 0.05 (low) to 0.29 (moderate) (Schöpke et al., 2015; Van der Waaij et al., 2005). Oberbauer et al. (2013) reported higher estimates of heritability of risk for DD along with other foot wart disorders of 0.30–0.40. Van der Linde et al. (2010) pointed out that, because the repeatability of phenotypic measures on claw disorders is low, the accuracy of EBV can be increased by taking repeated observations. Based on results of conventional models, they argued that an annual genetic response of −0.2 per cent point in the prevalence of DD is possible.

### 1.3 Aim

Here, we compare an analysis of disease status to an analysis of infection events for DD in Dutch dairy cattle, aiming to better understand the origin of the genetic variation in DD. We will use GEBV and cross‐validation to study the differences in both traits. As argued above, the analysis of disease status captures a combination of genetic variation in individual susceptibility and in the recovery of individuals from being infected. The analysis of infection events captures genetic variation in susceptibility only and is founded in epidemiological theory. The comparison of results for both traits, therefore, gives insight into the origin of genetic variation in disease status (i.e. susceptibility versus recovery), which has received very little attention in livestock genetic improvement.

## 2 MATERIALS AND METHODS

### 2.1 Data sampling

The data used in this study were collected from November 2014 to April 2015. The cows were just moved indoors before the start of this observation period. Observations on the DD‐status of the hind feet of individual cows were recorded during that period from 12 dairy farms from across the Netherlands, which previously had been reported endemic for DD with mean herd‐level prevalence of at least 20%. The number of cows per farm ranged from 88 to 189, and DD status of cows was recorded (in principle) at 11 observation moments per farm (Table 1). These 11 observation moments correspond to 10 observation intervals, during which cows could become infected or recover. The 11 observation moments were spaced bi‐weekly to accurately document the changes in DD‐status over time.

**TABLE 1:** Number of animals enrolled, number of observations and intervals between observations in the study per farm

Farms	No. of cows on farm	No. of observations/farm	Average Δt (days)^¶^
A	134	11	14
B	105	11	14
C	159	11	14
D	118	11	14
E	102	11	13.6
F	133	10	15.6
G	100	11	14
H	189	11	14
I	104	11	14
J	88	11	14
K	130	9	14
L	151	11	13.9
Total	1513	129	‐

^¶^ Standard deviation of interval between observations averaged over all farms was ±1 day.

The disease status of both hind feet was recorded prior to milking in the milking parlour. The detailed protocol for this procedure can be found in Biemans et al. (2018). In short, DD‐status was recorded using the M‐scoring technique, named after Mortellaro, following Cheli and Mortellaro (1974); see also (Berry et al., 2012; Döpfer et al., 2012; Relun et al., 2011). This system has 6 levels, M0 through M4.1. M0 is when clinical DD is absent, M1 is an acute form with a lesion <2 cm, M2 is a late acute form with a lesion larger than 2cm, M3 is an intermediate stage with a scab covering the lesion, M4 is a chronic stage with irregular skin (dyskeratosis) or superficial proliferation on top of an old lesion, and M4.1 is a special case of M4 where in addition to dyskeratosis there is another M1 like acute lesion.

Each animal had on average 8.7 records on DD status, with a target of 11 observations per animal throughout the data collection period. (The average number of observations per cow is smaller than the target of 11, because DD status was not recorded for dry cows.) Other factors that may affect the prevalence of DD on farm were recorded also (Table 2). These included the herd of the animal, animal ID, pedigree in the form of dam ID and sire ID, the genotype of the animal, birth date of the animal, coat colour of animal, breed of animal, parity, most recent date of calving, regular footbath strategy at the farm (present/absent), manure scraper (present/absent) and days in milk (DIM) at the moment of DD‐recording. Out of the 1513 animals that had phenotypic records, 1,401 animals were genotyped by the Dutch‐Flemish cattle breeding cooperative (CRV). Genotyping was done with the Eurogenomics™ 10 k SNP chips. The 10 k genotypes were imputed to ~80 k by CRV, using their standard imputation protocol. All cows were of the Holstein Friesian breed.

**TABLE 2:** Phenotypic data in 2014–2015*

Data header	Description	Levels
Animal	Official ID/tag number, for example NL882123242	1513
Farm	Farm code (A‐L)	12
Sire ID	Tag/ID of Sire	52
Dam ID	Tag/ID of Dam	128
Breed	Breed and its percentage, for example HF6ONB2 (meaning 6/8 HF)	18
Birth‐Date	Date of Birth	‐
Colour	Colour of cow	4
Foot	Hind feet (left and right)	2
Manure scraper	Present/Absent (1/0)	2
Grazing	Present/Absent (1/0)	2
Footbathing	Present/Absent (1/0)	2
Genotyped	Done/not Done (1/0)	2
Lactation	Parity/Lactation number	11
Calving Date	Date of last calving	‐
Score 1−11	M scores for observation moments 1–11 (10 intervals)	6
PH	Proliferative/Hyperkeratotic lesion	2

* Collected by Biemans et al. (2018).

### 2.2 Data restructuring and editing for the analysis of disease status

For the analysis of disease status, we used all records on disease status available in our data set, resulting in 12,195 records on 1,401 cows at 12 farms. M‐scores per foot were converted to binary format (0/1), where an M0 score was converted into 0, while feet with M1, M2, M3, M4 or M4.1 were converted into 1. Next, the scores of the two‐hind feed at an observation moment were averaged, so that animals were scored as y_DSM_ = 0, 0.5 or 1, 0 indicating two non‐infected feet, 0.5 indicating one infected foot and 1 indicating two infected feet. Thus, an animal had up to 11 observations, one for each of the 11 observation moments (“time of observation”, TO). Parity was expressed in 5 classes: 1 through 4, and greater than or equal to 5. Days in milk was converted to months in milk (MIM). All the above data restructuring were done using the R programming software R‐studio (R Core Team, 2013) and VBA macros for MS‐Excel 2013.

### 2.3 Data restructuring and editing for the analysis of infection events

For the analysis of infection events, only data on individuals that had at least one non‐infected foot at the beginning of an observation interval are informative (only those individuals can become infected). Hence, while we used all informative records from the full original data set, the number of records is smaller for the analysis of infection events than for the analysis of disease status. Thus, only animals with at least one non‐infected foot at the previous observation moment were included in the dependent variable for an observation interval. The resulting total number of records for the IM was 6,099, which is only half of that for the DSM. The M‐scores were converted to cases per foot. These cases (C) were considered as “successes” in binomial trials, with binomial total *F* = 1 or 2 non‐infected feet at the start of the observation interval. Hence, the resulting phenotypic measure (y_IM_ = C/F) was either 0, 0.5 or 1, with a binomial total of *F* = 1 or 2 susceptible feet. For example, an animal with two non‐infected feet at the previous observation moment (TO) and a single infected foot at the current observation moment had a y‐value of 1 infected foot/2 feet = 0.5, and *F* = 2. Parity was defined in the same way as for the DSM but with fewer factor levels.

Genotype data were filtered as described in detail in Biemans et al. (2019). Only SNPs with a minor allele frequency >0.025, and deviation of frequency from HW equilibrium <0.15, and a missing rate less than 5% were included. The remaining 75,904 SNPs (post‐filtering) were used to construct the genomic relationship matrix, which was used in both the DSM and the IM.

### 2.4 Disease status model (DSM)

To identify the fixed effects to be used in the DSM, we first fitted a univariate linear model without random effects for farm, parity, months in milk (MIM), time of observation (TO) and breed of the animal. We included fixed effects with a *p*‐value < 0.10 (Table 3).

**TABLE 3:** Univariate analysis of Significant factors of the disease status model (DSM)

Factor	*F*‐statistic	*p*‐Value^±^
Farm	39.553	<2.20E‐16
Parity	299.43	<2.20E‐16
MIM*	7.5075	0.006153
Time of Observation (TO)	7.2816	1.36E‐11
Breed	7.0391	<2.20E‐16

* Months in milk was evaluated along with its quadratic and cubic form.

^±^ *p*‐values in this table were tested against *p* = .1 for univariate analysis. All values were highly significant.

Interactions between significant fixed effect terms from Table 3 were tested, but had *p*‐value > 0.10, and were thus omitted from the model, except for the Farm*TO interaction which was included as a random effect (see below).

The final model was built with stepwise addition of fixed and random effects. Random effects of farm by observation interaction and farm by parity interaction were included to correct for the random variation from sampling duration and state of animals from particular farms due to their managemental protocols. A random non‐genetic animal effect (a so‐called permanent effect of the individual) was included to account for the repeated measures on same animal and its biological disposition. A random genetic effect was included with a genomic relationship matrix (**G**) constructed according to method 1 of (VanRaden, 2008), calculated using the calc_grm software (Calus and Vandenplas (2013). The final DSM was

(1) $$y_{\mathrm{DSM}}=X\beta +Z_{1}u_{1}+Z_{2}u_{2}+Z_{3}u_{3}+\;Z_{1}a+\;\epsilon$$

***y***_DSM_ is a vector of observations on the disease status/score of both feet of the animals, *y*_DSM,i_ = 0, ½ or 1, ***X*** is the known design matrix for all fixed effects found significant (Table 3) in univariate analysis including farm, parity, months in milk (MIM), TO (time of observation) and breed, **β** is the unknown vector of fixed effects, ***Z***_1_ and ***Z***_2_ and ***Z***_3_ are known design matrices for the animal, farm*TO and farm*parity, respectively,$u_{1}$, $u_{2}$and $u_{3}$ are unknown vectors of independent random effects of the animal (non‐genetic), farm*TO and farm*parity, respectively, and a is the unknown vector of random animal genetic effects (genomic breeding values), with $\sigma_{a}^{2}$is the additive genetic variance. ϵ is the vector of independent residuals. The total number of observations for the DSM was 12,195.

We also fitted the DSM with a pedigree‐based (based on 4 generations) relationship matrix (**A**), using $a∼N(0,{A\sigma }_{a}^{2})$, to compare the additive genetic variance and heritability between models with genomic versus pedigree‐based relationships.

The variance components and GEBV were estimated using the ASREML‐w software (Gilmour et al., 2008). The GEBV from the DSM were interpreted as the change in the probability of an animal (i) to be infected (y_DSM,i_ = 1) due to its genotype. For example, an animal with a GEBV of 0.1 will have a 10 per cent point higher probability of being infected due to its genotype than the average cow. The fixed effects and random non‐genetic effects are interpreted in the same way.

### 2.5 Infection model (IM)

We used the generalized linear model with mixed effects of Biemans et al. (2019) as the infection model (IM). The key difference between this IM and the above DSM is in the y‐variable. While the DSM fits individual disease status, the IM fits whether or not a non‐infected individual becomes infected during an observation interval. Hence, with the IM, we model disease susceptibility, the propensity of an individual to become infected given that it was non‐infected at the previous observation moment.

To account for variation in exposure of non‐infected feet to infected feet of herd mates (“infection pressure”) between farms and periods (intervals between two observations), the fraction of infected feet in the farm*period was included in an offset in the IM. The IM was

(2a) $$\mathrm{cloglog}\left(P_{\mathrm{iklt}}\left(t\right)\right)=\;c_{0}+{\mathrm{Farm}}_{k}+\;{\mathrm{Parity}}_{l}+\;c_{1}MIM+\;{\mathrm{Period}}_{t}\;+{\mathrm{Farm}}_{k}*{\mathrm{Period}}_{t}+\;{\mathrm{PE}}_{i}+A_{i}\;+\log\left(\frac{E\left(t\right)+\;I_{\mathrm{tot}}\left(t\right)}{N\left(t\right)}\Delta t\right)$$

where <cloglog> is the complementary log‐log link function (McCullaugh & Nelder, 1989), $P_{\mathrm{iklt}}\left(t\right)$ is the probability that a foot gets infected between time *t*‐1 and *t*, *c_0_* is an intercept, *Farm* is the fixed effect of the farm, *Parity* is the fixed effect of parity with three levels with 1, 2 > 2. $c_{1}$ is the regression coefficient of MIM, ${\mathrm{Period}}_{t}$ is the fixed effect of time period with 10 periods (intervals) resulting from 11 times of observation, ${\mathrm{Farm}}_{k}*{\mathrm{Period}}_{t}$ is the random interaction term of farm and period, ${\mathrm{PE}}_{i}$ is the random non‐genetic animal effect accounting for repeated observations on the same animal (also known as “permanent environment”), and $A_{i}$ is the random genetic animal effect, and the last term is the known offset. The offset $\log\left(\frac{E\left(t\right)+\;I_{\mathrm{tot}}\left(t\right)}{N\left(t\right)}\Delta t\right)$ was included to account for the infection pressure coming from the infected cows at the start of the observation interval, for the effect of infectious material accumulated in the environment, and for the length of the observation interval (Δ*t*; $I_{\mathrm{tot}}\left(t\right)$ is the total number of infected claws at the start of the observation interval, and E(t) represents the infection pressure from the environment (see Biemans et al. (2019) for details). Note that Equation 2a does not include genetic effects of herd mates on the record of the individual, meaning we ignored potential genetic variation in infectivity.

Because the y‐variable in the IM measures whether or not an individual becomes infected given exposure, the ${\mathrm{PE}}_{i}$ and $A_{i}$ in the IM are measures of the susceptibility of the animal. Specifically, Biemans et al. (2019) show that the IM yield predictions on the log scale of susceptibility, so that the breeding value of individual *i* for susceptibility follows from

(3) $$A_{\gamma ,i}=e^{A_{i}}.$$

The $A_{\gamma ,i}$ from Eq. 3 is the genetic component of the susceptibility $\gamma_{i}$ of individual *i*, as defined in Equation 3 of Appendix S1. This breeding value can be interpreted as an odds ratio. For example, an animal with $A_{\gamma ,i}$ = 3 is 3 times more likely to get infected compared to an average animal (which has $A_{\gamma ,i}$ = 1) given the same exposure in both. To calculate this heritability, we used a residual variance of π^2^/6 on the liability scale, which is the variance of the standard Gumbel distribution. We took this approach because a generalized linear model such as the IM with a complementary log‐log link function is equivalent to threshold model with a Gumbel distribution of the residual on the underlying scale (Nakagawa et al., 2017). This equivalence is similar to that of a generalized linear mixed effects model with a probit‐link function and a threshold model with a normally distributed residual.

The above model was fitted in ASREML‐ w (Gilmour et al., 2008) with generalized linear mixed models procedure. The generalized linear mixed model procedure in ASREML‐w uses the PQL algorithm (Penalized Quasi‐Likelihood, Breslow and Clayton (1993)) which is known to give biased estimates for the variance parameters (See Discussion). The difference between the DSM and the IM is summarized in Table 4.

**TABLE 4:** Differences between the disease status model (DSM) and Infection model (IM)

Model	Characteristics	Y‐variable
Disease status model (DSM)	Linear mixed model for disease status at an observation moment	Disease status score of 0, 0.5, 1 for disease present in no feet, 1 foot and 2 feet respectively
Infection Model (IM)	Generalized linear mixed model for new infection during an observation interval	New cases per foot (0,1)

### 2.6 Differences in DSM and IM

For the comparison of the results for the two traits, we focussed on four variables, the two GEBV from both models and the two y‐variables from both models. Hence, to compare and interpret differences between the analysis of the two traits, we considered all six pair‐wise correlations.

For the comparison of GEBV and y‐variables, we conducted 12‐fold cross‐validation (Kohavi, 1995). The 12 farms were the subset factor for both the DSM and the IM. In each of the 12 cycles, phenotypes of animals on one farm (test set) were left out from the analysis and the models were trained on the remaining 11 farms (reference set). Phenotypes of the test set where then predicted and compared to the actual observations for this set. For both the DSM and IM, we measured accuracy as the weighted correlation between predicted phenotypes and corrected phenotypes, weights being the number of observed records for an individual. We measured bias as the weighted regression coefficient of observed on predicted records.

For the DSM, the observed phenotypes were corrected for non‐genetic effects by fitting a linear model with only the fixed effects of Eq. 2; *y = FE* *+ e*. The residuals of this model were averaged over the 11 times of observations (TO), and the average was used as the corrected phenotype,

(4) $$y_{c}=\overset{‐}{y‐FE}$$

The predictive ability of the DSM was measured by the correlation between *y*_obs_ and the predicted GEBV,

(5) $$r_{GEBV,\;\;\;y_{c}}=\;\frac{\sigma_{GEBV,\;\;\;y_{c}}}{\sqrt{\sigma_{\mathrm{GEBV}}^{2}\sigma_{y_{c}}^{2}}}$$

For the IM, the cross‐validation procedure is not straight forward because the correlation between predicted and observed records depends on the value of the fixed effects (Biemans et al., 2019). This dependency occurs because the cloglog link function is non‐linear. For this reason, the GEBV from the IM were not directly compared with the phenotypes of the test set, but both predicted and observed records were transformed to a “standard herd”, following Biemans et al. (2019). Correlations between predicted and observed records were calculated using these transformed records. This procedure is described in full detail in Biemans et al. (2019).

## 3 RESULTS

### 3.1 Descriptive statistics

We calculated the mean prevalence of DD for each farm averaged over times of observations (Table 5) and for each scoring time of observation (TO) averaged over farms (Table 6). The overall average prevalence was 62.88% (±6.8), with a range of 49.38% (±2.8) to 77.7% (±5.4) between farms.

**TABLE 5:** Average prevalence by farm (at least 1 foot infected^±^)

Farm	Prevalence (%)	*SD* * (%P)
A	77.70	5.4
B	55.81	7.5
C	49.38	2.8
D	58.70	5.0
E	63.64	5.0
F	59.35	10.0
G	65.30	8.0
H	63.80	6.0
I	60.37	5.0
J	66.07	10.7
K	63.66	9.6
L	70.82	7.1
Average	62.88	6.8

^±^ An animal was counted as infected when at least one of its hind feet was infected.

* *SD* is the standard deviation of Prevalence among the 11 scoring observations.

**TABLE 6:** Average prevalence by time of observation^±^

Times of observation (TO)	Prevalence (%)*	*SD* (±%)
1	56.93	4.59
2	60.79	4.95
3	63.48	4.88
4	60.37	4.89
5	65.57	4.75
6	69.67	4.60
7	64.63	4.78
8	60.62	4.89
9	60.55	4.89
10	64.64	4.78
11	63.63	4.81
Average	63%	4.83

^±^ At least 1 foot infected per animal (total 2 hindfeet).

* Point prevalence is averaged over all 12 farms for each observation.

### 3.2 Disease status

**FIGURE 1:**
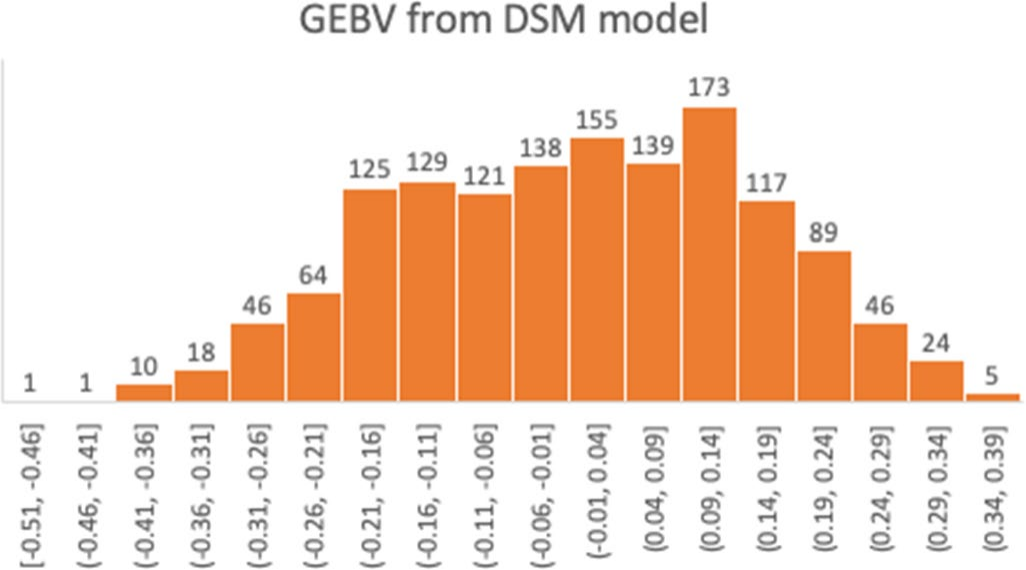
GEBV estimates of animals from DSM

The estimated additive genetic variance was 0.052 (±0.008), phenotypic variance was 0.19 (±0.006), and heritability (σ^2^_a_/σ^2^_p_) was 0.268 (±0.036; Table 7a). All random effects were significant (σ^2^/*SE* > 2), except for farm*parity. The fixed effects of TO, lactation levels and months in milk were significant, but farm and breed did no longer have a significant effect on the disease status in the model including random effects (Table 7b). The GEBV from the DSM were between −0.50 and 0.37, with a mean of 0. Hence, they cover 87% of the 0–1 range, indicating very large genetic differences among individuals (Figure 1).

**TABLE 7a:** Variance components of DSM model

Model term	Variance	*SE*	Var/*SE*
Animal genetic variance	0.0518	0.008	6.51
Animal (non‐genetic)	0.0725	0.006	11.82
Farm*observation	0.0033	0.0005	6
Farm*Parity	0.0039	0.002	1.93
Residual	0.0641	0.0009	73.07
Phenotypic variance	0.192	0.0059	
Heritability	0.268	0.0362	

**TABLE 7b:** Fixed effects of DSM

Model term	Df^#^	F‐stat*	*p*‐value
Farm	11	1.96	0.053
Observation	10	5.07	<0.001
Breed	12	0.89	0.562
Parity	4	17.76	<0.001
MIM	1	11.75	<0.001

^#^ Df stands for degrees of freedom for approximate *F* test.

* F‐stat stands for approx. *F* statistic.

In the analysis of the model based on pedigree, the estimated additive genetic variance was 0.0514 (±0.011), phenotypic variance was 0.192 (±0.0056), and heritability was 0.268 (±0.05). There was no significant difference in the heritability estimates between the model based on genomic relationship and the model based on pedigree.

### 3.3 Infection events

The estimated heritability from the IM was 0.16. Note that the heritability estimate of 0.16 is on the liability scale, similar as “underlying heritability” in the threshold model of Dempster et al. (1952). For the complementary log‐log link function, the transformation of underlying heritability to observed heritability is unknown to our knowledge. Hence, we cannot translate the estimate to the observed binary scale. The fixed effects of Parity (*p* < .001), months in milk (MIM) (*p* < .001) and period (*p* = .039) were significant (Table 8). Farm was not a significant fixed factor, probably because the offset accounted for most of the variation among farms. From the random components, farm by period interaction, animal genetic component and animal non‐genetic component were all significant.

**TABLE 8:** Results of IM model for fixed and random effects

Model term	*df* ^	F‐stat¹	*p*‐value
mu	1	2065.83	<0.001
Parity	2	26.49	<0.001
MIM	1	38.57	<0.001
Period	9	2.08	0.039
Farm	11	1.09	0.371

Model term	Variance	*SE*	Var/*SE*
Farm*Period	0.261	0.05	5.22
Animal genetic variance	0.555	0.141582	3.92
Animal (non‐genetic	0.949	0.13	7.3
Heritability (approx.)	0.16		‐
Residual*	π^2^/6	NA	NA

^ Df: Degrees of freedom for approximate F‐test

* Residual variance on liability scale is π^2^/6

¹ F‐stat stands for approx. F statistic.

Variance components from the IM analysis are known to be biased, because the IM is fitted using the PQL‐algorithm which uses the BLUEs and BLUPs in the iteration. The bias depends on the number of fixed effects and on the number of records per random effect. For simple models, the bias it is approximately −1/n_records_. We had an average of 8.7 observation periods per cow, and in ~60% of cases, a cow had at least one susceptible foot, yielding ~5.22 records per cow. Hence, this would suggest a bias of about −19%. However, because of genetic relationships between cows, the bias is probably less than −1/n_records_ (Engel et al. (1995); Bas Engel, personal communication).

**FIGURE 2:**
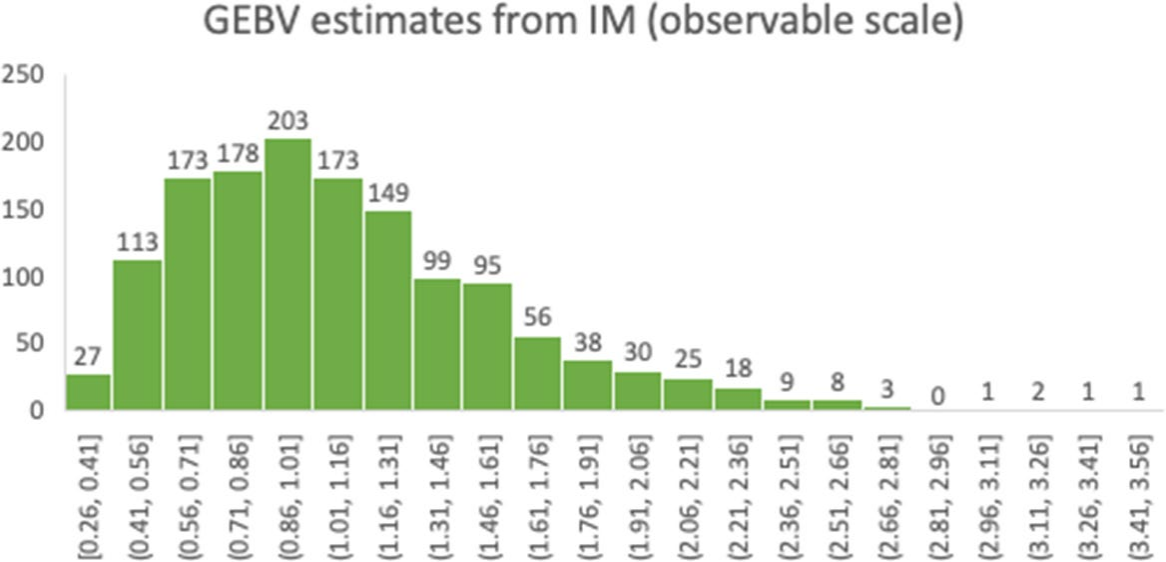
GEBV estimates from IM on observable scale

The GEBV estimates from the IM represent the logarithm of the animal genetic effect on relative susceptibility (Biemans et al. 2019). These estimates were exponentiated to obtain the GEBV on relative susceptibility ($A_{\gamma }$). The resulting values were centred around 1 and ranged from 0.26 and 3.43 (Figure 2). These GEBV can be interpreted as relative risk. For example, the cow with 3.43 GEBV has 3.43 times higher probability (per unit time) to become infected than an average cow with average risk of 1. So, in general, the top (3.43) and the bottom (0.26) individuals differ by a factor of ~13 in probability to get infected. Hence, similar to the DSM, results from the IM suggest very large genetic differences among individuals.

### 3.4 Differences in DSM and IM

#### 3.4.1 dsm

**FIGURE 3:**
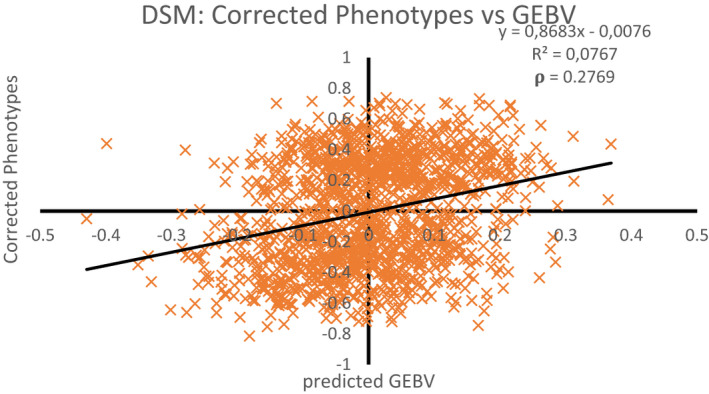
Corrected Phenotypes and GEBV predictions from DSM model. The y‐axis shows phenotypes corrected for fixed effects and x‐axis GEBV from Cross‐validation. ρ is a weighted correlation coefficient

The Pearson's weighted correlation coefficient between corrected phenotypes per animal and its GEBV (predicted) was 0.28 (Figure 3), and Spearman's rank correlation coefficient was 0.27. The regression coefficient of corrected phenotypes on GEBV predictions was 0.87, indicating that the GEBV overpredict the differences in true breeding value by ~15%. The approximate accuracy of GEBV was calculated as $r_{\hat{A}A}=\;r_{\hat{A}\bar{P}}/h_{\bar{P}}$, where $\hat{A}$ is the GEBV, *A* is the true breeding value, $\bar{P}$ is the corrected phenotype, *h* is the square root of the heritability of the corrected phenotype, and *r* is Pearson's correlation coefficient. The corrected phenotype was an average of on average *n* ≈ 8.7 records on an individual. The heritability of the corrected phenotype was calculated as

$$h_{\bar{P}}^{2}=\frac{\sigma_{a}^{2}}{\sigma_{a}^{2}\;+\;\sigma_{u_{1}}^{2}\;+\;\sigma_{u_{2}\;}^{2}/n\;\;+{\;\;\sigma }_{u_{3}}^{2}{\;+\;\sigma }_{\epsilon \;}^{2}/n}=\;0.381$$

In the denominator of this expression, we did not apply averaging to $\sigma_{u_{3}}^{2}$ because most records on an individual came from the same parity. The resulting accuracy was 0.28/√0.381 ≈ 0.45. This means that the DSM‐based GEBV predict the true breeding value for an individual's disease state with an accuracy of ~45%.

### 3.5 im

**FIGURE 4:**
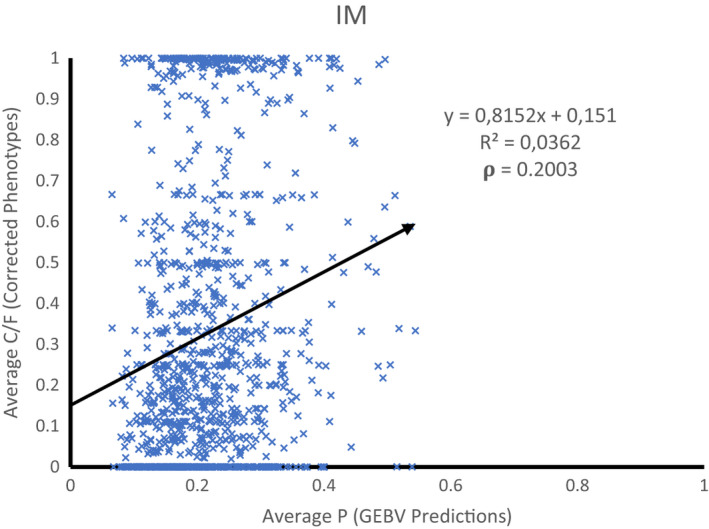
Weighted Correlation ρ (weights = #period animal was susceptible) between Corrected phenotypes of IM and GEBV predictions from cross‐validation

The weighted Pearson's correlation (ρ) coefficient between corrected phenotypes and predicted probabilities was 0.20 (Figure 4). Note that observation and predictions of IM refer to whether (1) or not (0) a susceptible individual becomes infected, not to the disease status as seen in the DSM. The weighted regression coefficient of the corrected phenotypes on the predicted probabilities was 0.81, indicating an overprediction of differences in true breeding value by ~23%. We cannot calculate an approximate accuracy here, because the heritability of the trait on the observed scale is unknown (see Methods).

Table 9 shows the correlations between the predicted and observed (corrected) phenotypes of both the DSM and the IM.

**TABLE 9:** Pearson's correlation between cross‐validation outcomes of two models^±^

	IM_predicted	IM_phenotype	DSM_predicted	DSM_phenotype
IM_predicted	1	0.20	0.63	0.09
IM_phenotype		1	0.22	0.75
DSM_predicted			1	0.28
DSM_phenotype				1

^±^ IM_predicted, predicted probabilities from IM cross‐validation; IM_phenotype, corrected phenotypes from IM cross‐validation; DSM_predicted, predicted probabilities (based on GEBV) of DSM cross‐validation; DSM_phenotype, corrected phenotype from DSM cross‐validation.

The phenotypes used in the DSM versus IM correlated by 0.753, indicating that disease status (the DSM y‐variable) is positively correlated with, but clearly different from, becoming infected or not (the IM y‐variable). Similarly, the GEBV from both models are positively correlated (0.629), but the correlation is clearly different from 1. Hence, both models capture different traits. Overall, the correlation for DSM predictions is higher than for IM predictions, which probably reflects the substantial difference in the number of informative records. The IM better predicts disease transmission than disease status (0.20 versus 0.09), while the DSM better predicts disease status than disease transmission (0.28 versus 0.22).

## 4 DISCUSSION

### 4.1 Brief summary

The main purpose of this study was to present and compare estimated genetic parameters and breeding values for the binary disease status and infection events. Disease status was analysed with a linear mixed model (DSM), while infection events were analysed with a generalized linear mixed model. For both traits, we used all informative records from a data set on the endemic claw disease DD in dairy cattle. Both traits showed significant and substantial additive genetic variance and a significant correlation between the GEBV of the two traits and observed phenotypes in the cross‐validation. These results demonstrate the presence of heritable variation, both in DD disease status and in susceptibility to become infected with DD.

### 4.2 Results for disease status

The estimated heritability of binary disease status from the DSM was 0.27, which is in the upper range of values found in the literature (0.05–0.29; (Schöpke et al., 2015; Van der Linde et al., 2010; Van der Waaij et al., 2005)). This relatively high value may reflect the selection of farms that were known to be endemic for DD, and the fact that our y‐variable was the average of the disease status of both hind feet of a cow, rather than a single binary record. In our data, prevalence was 63%, which is higher than in the studies of Van der Waaij et al. (2005; 21.7%) and Schöpke et al. (2015; 17%). Hence, this difference in prevalence may explain the relatively high heritability found here (Dempster & Lerner, 1950).

Lactation level significantly affected DD status, and the estimates increased from lactation level 1–4, but then dropped for 5 or later lactations. This was in contrast with results of de Jesús Argáez‐Rodríguez et al. (1997) and Frankena et al. (1991), who found an increased risk for the 1st and 2nd parity, but lower risk in subsequent parities. The times of observation (TO) were also a significant fixed effect, and the 6th observation had a 14% higher estimate than the 1st observation. Since the data were collected in the winter of 2014 and the spring of 2015, we hypothesize that the absence of grazing has contributed to this pattern, in line with findings of Frankena et al. (1991) and Read and Walker (1998).

The cross‐validation of DSM resulted in a weighted correlation of ~0.28 between corrected phenotypes and predicted phenotypes based on GEBV. From the cross‐validation procedure, the accuracy of DSM‐GEBV was approximately 45%, which is reasonable. However, this result might be specific for our data set, which consisted of farms with a high prevalence of DD. The regression coefficient of corrected phenotypes on predicted breeding values in the cross‐validation was 0.87, which shows that the breeding values were slightly overestimated. Nevertheless, when removing bias by multiplying GEBV by 0.87, the range of GEBVs in Figure 1 is still substantial. Hence, despite some overestimation of GEBV, results of the DSM suggest large genetic variation in DD status of dairy cattle in the Netherlands.

### 4.3 Model assumptions and interpretation: DSM

In the DSM, the phenotype and the genetic factor have a linear relationship, and random effects follow a normal distribution. These assumptions are clearly violated given the binomial nature of the data with binomial total being 1 or 2. However, the practical relevance of this violation may be limited as benefits of using threshold models over linear models are relatively small for the estimation of breeding values (e.g. see Ramirez‐Valverde et al. (2001)). More importantly, the DSM disregards the transmission and recovery dynamics of infectious diseases, which hampers the biological interpretation of GEBV from the linear model. Bijma (2020; Equation 4.4) argues that, for the simplest model of an endemic disease in which there is no distinction between infected and infectious individuals, the breeding value of the DSM is a function of the rate at which an individual recovers from being infected (α), the transmission rate parameter for the individual (β), and the prevalence of the disease,

(6) $$A_{i}=\frac{1/\alpha_{i}}{1/\alpha_{i}\;\;+\;1/\left(\beta_{i}P\right)}$$

The numerator of this expression (Equation 6) is the average time individual *i* stays in the infected state (1) while the denominator is the average duration of a full cycle from susceptible to infected and back to susceptible. This expression relates the breeding value from the linear model to epidemiological parameters and shows that an individual's breeding value for disease status includes the genetic component of its susceptibility ($\beta_{i}$) and its ability to recover from being infected ($\alpha_{i}$), where the relative contribution of both components depends on the prevalence of the disease. Note that the GEBV from the DSM predict individual disease status in a population where the prevalence (and thus the “infection pressure”) is the same as in the population from which the GEBV are estimated. The GEBV do not predict response to selection at the population level, because the DSM may capture recovery from infected but non‐infectious states and because it ignores indirect genetic effects originating from transmission dynamics (Bijma, 2020).

### 4.4 Results for infection events

The IM developed by Biemans et al. (2019) was re‐applied in this study for comparison with the DSM. This particular model had fixed effects, a genetic effect of the animal, and non‐genetic random effects for farm, animal and period of observation. The variance components in the IM are biased due to the PQL algorithm implemented in ASREML. Based on findings for simple models, this bias is expected to be smaller than 19%, but precise quantification would require stochastic simulations (personal communication Bas Engel). Heritability was estimated to be 0.16. This value is difficult to compare with literature results, since we are not aware of any other estimates for the heritability of susceptibility based on a IM with a complementary log‐log link function.

### 4.5 Model assumptions: IM

To preserve the model developed by Biemans et al. (2019) for the analysis of infection events, the number of levels in the model factor for parity was different compared to DSM. The IM was fitted to the number of cases (feet getting infected) over the number of feet that were susceptible at the start of a particular observation period. Because of this, the number of records for the IM differed from the DSM. The GEBV of the IM is an estimate of the susceptibility of an animal; in contrast to the DSM, it does not include a component due to recovery. However, even with variation in susceptibility only, there will still be some difference in GEBV between the two models, since they have different assumptions for the distribution of the data and include different model terms. The GEBV from the IM are on the log scale and were exponentiated to obtain GEBV on the scale of susceptibility (γ). The resulting GEBV are the approximate odds for a susceptible animal to become infected, relative to the average animal which has γ = 1. Hence, in contrast to the DSM, the IM does not capture genetic variation in the ability of individuals to recover from being infected (α).

Moreover, both the DSM and the IM did not consider potential genetic variation in the propensity of (infected) herd mates to infect the focal individual. The IM accounted for the number of infected herd mates by including a known offset equal to $\log\left(\frac{E\left(t\right)+\;I_{\mathrm{tot}}\left(t\right)}{N\left(t\right)}\Delta t\right)$. Fitting of an offset is common in GL(M)M in epidemiology, but might have removed some genetic variation, because an offset is treated as a known effect, and thus not estimated simultaneously with the genetic effects. While the DSM does not explicitly account for the number of infected herd mates, their effect is captured by the fixed Farm effect and the random farm*period effect, at least partly.

The IM used here is fundamentally different from the classical threshold model (Dempster et al., 1952; Gianola, 1982). While both models connect an observed binary y‐variable to an underlying linear predictor using a link function, the IM used here is founded in epidemiological principles and yields estimates for the susceptibility of individual to become infected given it was non‐infected at the previous observation moment, whereas the ordinary threshold model specifies an underlying normally distributed liability without reference to disease transmission dynamics. Specifically, we analysed transmission events (*i.e*. animals becoming infected), whereas the ordinary threshold model is typically used to analyse the binary disease status observed at any time point.

### 4.6 Time‐dependent GEBV from IM

Anacleto et al., (2015) argued that repeated observations on the disease status of animals may improve the accuracy of GEBV for susceptibility derived from the IM. In this case, the GEBV for susceptibility depends on the amount of time in between two observations relative to the duration of a disease status. When observations are far apart in time, changes in disease status may be missed, resulting in reduced accuracy and potential bias in GEBV. Moreover, IM does not include the time of infection (1/recovery rate) parameter and the true duration of infection is unknown (Biemans et al., 2019).

To account for the systematic changes in the probability of a susceptible foot to get infected in the given observation interval (period) and on the given farm, a random effect for farm X period was included in the model (see Material and Methods). This systematically controlled for effects of the period (duration) on the given farm from which the animal (and subsequently the susceptible hind foot) originates.

### 4.7 Breeding against Infected versus Infectious

The relevance of a distinction between infected individuals (having disease status 1) and infectious individuals (being able to infect others) was a key motivation for this study. In the Introduction, we stated that the prevalence of an infectious disease depends on the duration that individuals are infectious, while the duration that they are infected is irrelevant. In Appendix S2, we illustrate that, with respect to recovery, selection against infected but non‐infectious individuals does not change the prevalence of an infectious disease. Hence, with respect to genetic selection for recovery, this example illustrates that such selection should target recovery from being infectious, rather than from being infected. Both may of course overlap to a larger or smaller degree, which will depend on the specific disease.

## 5 CONCLUDING REMARKS

The above comparison of the DSM and the IM shows that the DSM captures both the disease susceptibility of an individual and its ability to recover from being infected, whereas the IM captures susceptibility. The difference in GEBV of both models suggests that genetic variation in disease status for DD originates not only from genetic variation in susceptibility, but also from genetic variation in recovery. With genetic variation in recovery, the distinction between infected individuals and infectious individuals becomes relevant for response to selection if selection is focused on recovery along with susceptibility. Hence, a genetic analysis of recovery events would be interesting, particularly when data are available on the infectiousness of the infected individuals. A joint analysis of susceptibility (e.g. with the IM used here) and recovery of individuals from being infectious would provide insight into the genetic effects underlying the prevalence of an infectious disease, and thus in potential response to combined selection on susceptibility as well as recovery.

## Supporting information

Appendix S1

Appendix S2

## Data Availability

The data and the code for statistical analyses that support the findings of this study are available from the corresponding author upon reasonable request.
