# Supplementary material for: On the origin of the genetic variation in infectious disease prevalence: Genetic analysis of disease status versus infections for Digital Dermatitis in Dutch dairy cattle
Source: J Anim Breed Genet. 2021 Jun 9;138(6):629–42. doi: 10.1111/jbg.12635 (PMC8518086; doi:10.1111/jbg.12635)
Supplement: Supplementary file 1 — Appendix S1 [file JBG-138-629-s002.docx]

# APPENDIX 1: BASIC EPIDEMIOLOGY THEORY

According to well-established epidemiological theory, the prevalence of an infectious disease is determined by the so-called basic reproduction number (or rate), $R_{0}$ (Anderson & May, 1979). *R_0_* is the average number of animals that gets infected by a typical infectious individual over its entire infectious duration, in a fully susceptible population (*i.e.,* where all other individuals are non-infected). As common in epidemiology, we will use the term “susceptible” to indicate that an individual is non-infected and can in principle become infected; the term “susceptible” does not indicate any degree of disease susceptibility or resistance. In contrast to a breeding value, which is a property of a single individual, *R_0_* is a parameter of a population. For an endemic disease in equilibrium, the prevalence (P) follows from $R_{0}$ (Dietz & Heesterbeek, 2000)

$P=1-1/R_{0}$. **(Eq. 1)**

For example, when a typical infectious individual would infect three new individuals in a fully susceptible population (R_0_ = 3), then the expected (equilibrium) prevalence is 2/3, so that, on average, 66% of the individuals will be infected at any time. (The reason is that the equilibrium occurs when the effective reproduction rate, $R=R_{0}\left( 1-P \right)$, is equal to one, so that an infected individual is on average replaced by a single new infected individual. In this example, this happens when only 1/3 of the contact individuals is susceptible, so that 3 * 1/3 = 1). Thus, according to epidemiological theory, breeders that aim to reduce prevalence should focus on reducing $R_{0}$. ((Heesterbeek & Dietz, 1996); Equation 1 is an approximation when individuals vary in susceptibility; methods to account for such variation can be found in (Diekmann et al., 2012); this is beyond the scope of the current study).

R_0_ depends on the transmission rate parameter (β) and on the rate individuals recover from being infectious (the “recovery rate parameter”, α; Diekmann and Heesterbeek (2000)),

$R_{0}=\frac{\beta}{\alpha}$ **(Eq. 2)**

The transmission rate parameter β equals the average number of transmissions per unit of time due to a single infectious individual when all its contact individuals are susceptible, while the reciprocal of the recovery rate parameter, $1/\alpha$, equals the average duration of the infectious period. (When $\alpha$ varies over time, a weighted average can be taken; Diekmann and Heesterbeek (2000)). Hence, the product $\beta1/\alpha$ is the expected number of transmissions during the entire infectious period of the individual, which by definition is $R_{0}.$

In epidemiology, variation in disease “resistance” among individuals is commonly modelled as variation in their susceptibility ($\gamma$) to become infected. While both approaches are equivalent, modelling variation in susceptibility is more convenient and we will follow that approach. When individuals differ in their susceptibility to become infected, the rate at which individuals become infected varies among individuals. For this reason, we have to specify a transmission rate parameter for each individual (say i). Thus, for individual ‘i’, the transmission rate parameter becomes $\beta_{i}$ = $\beta$ *$\gamma_{i}$.

$\beta_{i}=c\gamma_{i}$ **(Eq. 3)**

where $\gamma_{i}$ is the (relative) susceptibility of animal *i*, and c is the average contact rate between the susceptible and infectious animals which results in an infection (the so-called “effective contact rate”). Thus, the mean value of $\gamma$ equals one, so that the mean $\beta$ equals the effective contact rate *c*. Hence, since prevalence depends on $R_{0}$, while $R_{0}$ depends on susceptibility, breeding for lower susceptibility is a means to reduce disease prevalence.

Given the role of susceptibility in disease prevalence, as illustrated above, new models have been developed to estimate the genetic variation in susceptibility and to compute genomic estimated breeding value (GEBV) for susceptibility (Anacleto et al., 2015; Anche et al., 2015; Biemans, 2018; Biemans et al., 2019; Lipschutz-Powell et al., 2012). These models are founded in epidemiological theory to capture the stochasticity of disease transmission and account for the fact that an individual’s disease status depends on the other individuals in the herd. For this reason, the GLM-versions of these models (Anche et al., 2015; Lipschutz-Powell et al., 2012) differ from the traditional GLM based on the threshold model (Dempster & Lerner, 1950; Gianola, 1982).

## Appendix References

Anacleto, O., Garcia-Cortés, L. A., Lipschutz-Powell, D., Woolliams, J. A., & Doeschl-Wilson, A. B. (2015). A novel statistical model to estimate host genetic effects affecting disease transmission. *Genetics, 201*(3), 871-884.

Anche, M. T., Bijma, P., & De Jong, M. C. (2015). Genetic analysis of infectious diseases: estimating gene effects for susceptibility and infectivity. *Genetics Selection Evolution, 47*(1), 85.

Anderson, R. M., & May, R. M. (1979). Population biology of infectious diseases: Part I. *Nature, 280*(5721), 361.

Biemans, F. (2018). Transmission of digital dermatitis in dairy cattle: population dtynamics and host quantitative genetics. *Wageningen University*.

Biemans, F., de Jong, M. C. M., & Bijma, P. (2019). Genetic parameters and genomic breeding values for digital dermatitis in Holstein Friesian dairy cattle: host susceptibility, infectivity and the basic reproduction ratio. *Genetics Selection Evolution, 51*(1), 67. doi:10.1186/s12711-019-0505-3

Dempster, E. R., & Lerner, I. M. (1950). Heritability of threshold characters. *Genetics, 35*(2), 212.

Diekmann, O., Heesterbeek, H., & Britton, T. (2012). *Mathematical tools for understanding infectious disease dynamics* (Vol. 7): Princeton University Press.

Diekmann, O., & Heesterbeek, J. A. P. (2000). *Mathematical epidemiology of infectious diseases: model building, analysis and interpretation* (Vol. 5): John Wiley & Sons.

Dietz, K., & Heesterbeek, J. (2000). Bernoulli was ahead of modern epidemiology. *Nature, 408*(6812), 513-514.

Gianola, D. (1982). Theory and Analysis of Threshold Characters 1. *Journal of Animal Science, 54*(5), 1079-1096.

Heesterbeek, J., & Dietz, K. (1996). The concept of Ro in epidemic theory. *Statistica neerlandica, 50*(1), 89-110.

Lipschutz-Powell, D., Woolliams, J. A., Bijma, P., & Doeschl-Wilson, A. B. (2012). Indirect genetic effects and the spread of infectious disease: are we capturing the full heritable variation underlying disease prevalence? *PLoS One, 7*(6), e39551. doi:10.1371/journal.pone.0039551
