# Supplementary material for: On the origin of the genetic variation in infectious disease prevalence: Genetic analysis of disease status versus infections for Digital Dermatitis in Dutch dairy cattle
Source: J Anim Breed Genet. 2021 Jun 9;138(6):629–42. doi: 10.1111/jbg.12635 (PMC8518086; doi:10.1111/jbg.12635)
Supplement: Supplementary file 2 — Appendix S2 [file JBG-138-629-s001.docx]

## **APPENDIX 2**

## Numerical Example

In a SEIS model, individuals move through the states susceptible (S), infected but not yet infectious (E), infectious (I) and back to S (Figure 1 of this appendix). In the simple deterministic version of this model, the transition rates are given by Brauer (1995).

$\frac{dS}{dt}=\alpha I-\beta IS/N$ **(Eq. 1a)**

$\frac{dE}{dt}=\frac{\beta IS}{N}-\varepsilon E$ (**Eq. 1b)**

$\frac{dI}{dt}=\varepsilon E-\alpha I$ **(Eq. 1c)**

where symbols in italics denote the number of individuals with the corresponding disease status, *N* the population size, and *β*, $\varepsilon$ and $\alpha$ the rate parameters (notation might differ from reference source). For example, if $\alpha=0.10/day$, on average 10% of the I-individuals move from the infectious state (I) to the susceptible state (S), and the mean duration of the infectious period of an individual equals $\frac{1}{\alpha}=10day$s. The equilibrium occurs when $\frac{dS}{dt}= \frac{dE}{dt}=\frac{dI}{dt}=0$. Solving the equations for this condition yields the equilibrium number of individuals of each state,

$S_{\infty}=N\frac{\alpha}{\beta}$ (**Eq. 2a)**

$E_{\infty}=NP_{\infty}\left( \frac{\alpha}{\varepsilon+\alpha} \right)$ **(Eq. 2b)**

$I_{\infty}=NP_{\infty}\left( \frac{\varepsilon}{\varepsilon+\alpha} \right)$ **(Eq. 2c)**

where $P_{\infty}=(N-S_{\infty})/N$, being the prevalence in the equilibrium. Note that $\frac{\alpha}{\beta}=1/R_{0}$ (Diekmann & Heesterbeek, 2000). These equations show that prevalence is determined by R_0_, while the distribution of infected individuals over the E and I classes is proportional to the relative magnitudes of $\alpha$ and $\varepsilon$. In the equilibrium, $\alpha I=\frac{\beta IS}{N}=\varepsilon E$, $\alpha I_{\infty}=\frac{\beta I_{\infty}S_{\infty}}{N}=\varepsilon E_{\infty}$ which is the number of transitions per day (the “turn-over rate”).

Now consider a population of $N=100$ individuals, with β = 0.3/day, ε = 0.2/day and α = 0.1/day. The equilibrium is given by $S_{\infty}=33.33$, $P_{\infty}=\frac{100-33.33}{100}=0.66$, $E_{\infty}=66.66 \times\frac{1}{3}=22.22$ and $I_{\infty}=66.66 \times\frac{2}{3}=44.44$. The mean durations of each state are $\frac{1}{\frac{\beta I}{N}}= 7.5 days$ for the susceptible period, $\frac{1}{\varepsilon}=5 days$ for the E-state, and $\frac{1}{\alpha}=10 days$ for the I state. Hence, the mean duration of a full cycle equals 22.5 days, and on average individuals stay infected for 15 days. Note that prevalence is also equal to the fraction of time individuals are infected, *P* = (5+10)/22.5 = 0.66. Moreover, on average, $\alpha I=\frac{\beta IS}{N}=\varepsilon E=4.44$ individuals per day move from one state to the next. Note that 22.5 x 4.44 = 100 = *N*.

Now suppose selection for recovery has shortened the E-stage to practically zero. The differential equation for *S* remains the same, that for *E* can be dropped, and

$\frac{dI}{dt}=\beta IS/N-\alpha I$. **(Eq. 3a)**

Solving the differential equations for zero equals

$S_{\infty}=N\frac{\alpha}{\beta}$ **(Eq. 4a)**

$I_{\infty}=N-S_{\infty}$ **(Eq. 4b)**

The equilibrium is given by $S_{\infty}=33.33$, $I_{\infty}=66.66$, and $P_{\infty}=0.66$. The mean durations of each state are 5 days for the susceptible period and 10 days for the I state. The mean duration of a full cycle, therefore, equals 15 days, and on average, $6.66$ individuals per day move from one state to the next. Hence, individuals now recover sooner, but prevalence stays the same.

This example, therefore, illustrates that simple selection against binary disease status does not necessarily reduce the prevalence of an infectious disease (note, prevalence is equal to the mean value of binary disease status), because the transmission dynamics may adapt. Reducing prevalence by selecting individual for faster recovery is effective only when it reduces the duration of the *infectious* period; a mere reduction of the duration of the infected period has no impact.

## Illustration of Breeding against Infected vs Infectious

The prevalence of an endemic infectious disease reaches an equilibrium value when an infected individual is on average replaced by a single new infected individual. In other words, when the effective reproduction number of the disease (R_E_) equals one. In a fully susceptible population, *i.e*., where all individuals are non-infected, the number of individuals that becomes infected due to a single infectious individual is by definition equal to *R_0_*, the basic reproduction number of the disease. While R_0_ is a fundamental parameter of a disease, the R_E_ is variable and depends on the fraction of susceptibles among the contact individuals in the population at any point in time;$R_{E}=\frac{S}{N}R_{0}$ (Dietz & Heesterbeek, 2000). Hence, an equilibrium occurs when $\frac{S}{N}R_{0}=1$. Thus, the equilibrium prevalence is given by $P=\frac{N-S}{N}=1-1/R_{0}$.


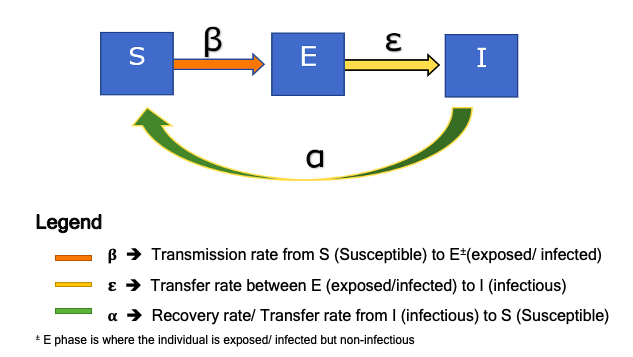


**Figure 1. SEIS model for transmission of endemic diseases**

Now consider an endemic infectious disease where individuals can be in one of three states: susceptible (S), infected but not yet infectious (E, from exposed), and infectious (I); S→E→I→S, see Figure 1. The rate at which individuals move from one state to the next is governed by three parameters, β, ε and α (see legend of Figure 1). The prevalence, i.e., the fraction of individuals that is infected, equals *P* = (*E*+*I*)/*N*, where *E* and *I* in italics denote the number of individuals with disease status E and I respectively, and population size equals *N* = *S* + *E* + *I*. Since the mean binary disease status equals *P* = (*E*+*I*)/*N*, selection aiming to reduce prevalence based on GEBV from the DSM targets both *E* and *I*, and thus may result in an increase of both *ε* and *α*, depending on which parameter shows (most) genetic variation. Suppose only ε shows genetic variation, so that continued selection might fully remove the E-state and the model would reduce to S→I→S. Since prevalence becomes *P* = *I*/*N*, this would at first glance seem to reduce the prevalence. However, the equilibrium prevalence still follows from $R=\frac{S}{N}R_{0}$ = 1. The fraction of susceptibles, therefore, still equals 1/R_0_, so that the prevalence $P=\frac{E+I}{N}=1-1/R_{0}$, irrespective of the distribution of infected individuals over the E and I states. Thus, selection against infected but non-infectious individuals (the E-class) does not change the prevalence of an infectious disease.

This apparent paradox occurs because removal of the E-state increases the rate at which individuals move through the S(E)IS cycle, and hence also increases the influx of new susceptibles. Consider, for example, a population of *N* = 100 members, with parameters *β* = 0.3/day, *ε* = 0.2/day and *α* = 0.1/day. This population has *R*_0_ = *β*/*α* = 3. In the endemic equilibrium *P =* $1-1/R_{0}$ = 2/3, S = 33.33, E = 22.22 and I = 44.44 (see Numerical Example in this appendix). Each day on average 4.44 individuals move from one state to the next (this is the same for all states because of the equilibrium). The average duration of the infected state (the sum of the I and E states) equals 15 days. Now suppose selection against being infected removes the E-state (meaning *ε* becomes very large). The new equilibrium is given by *P* = 2/3, *S* = 33.33 and *I* = 66.66, but on average 6.66 individuals per day move from one state to the next, and the average duration of the infected state equals 10 days. Hence, while the turn-over rate has increased, the prevalence of the disease remains unaffected. With respect to genetic selection for recovery, this example illustrates that such selection should target recovery from being infectious, rather than from being infected. Both may of course overlap to a larger or smaller degree, which will depend on the specific disease.

## Appendix References

Brauer, F., & Castillo-Chavez, C. (1995). Basic models in epidemiology. In *Ecological time series* (pp. 410-447). Springer, Boston, MA.

Dietz, K., & Heesterbeek, J. (2000). Bernoulli was ahead of modern epidemiology. *Nature, 408*(6812), 513-514.
